# Global research hotspots and trends of iodinated contrast agents in medical imaging: a bibliometric and visualization analysis

**DOI:** 10.3389/fmed.2024.1506634

**Published:** 2024-11-22

**Authors:** Yun Liu, Yonghai Dong, Fei Xie

**Affiliations:** ^1^Department of Imaging, Jiangxi Provincial People’s Hospital (The First Affiliated Hospital of Nanchang Medical College), Nanchang, Jiangxi, China; ^2^Jiangxi Provincial Key Laboratory of Major Epidemics Prevention and Control, Young Scientific Research and Innovation Team, Jiangxi Provincial Center for Disease Control and Prevention, Nanchang, Jiangxi, China; ^3^Guangdong Medical University, Guangzhou, Guangdong, China

**Keywords:** iodine contrast agents, medical imaging, bibliometrics, visualization, CiteSpace, VOSviewer

## Abstract

**Objective:**

This study employs bibliometric methods to explore the global research dynamics of iodine contrast agents in medical imaging. Through the visualization of knowledge maps, it presents research progress and reveals the research directions, hotspots, trends, and frontiers in this field.

**Methods:**

Using Web of Science Core Collection database, CiteSpace and VOSviewer were employed to conduct a visual analysis of the global application of iodine contrast agents in medical imaging over the past four decades. The analysis focused on annual publication volume, collaboration networks, citation characteristics, and keywords.

**Results:**

A total of 3,775 studies on the application of iodine contrast agents in medical imaging were included. The earliest paper was published in 1977, with a slight increase in publications from 1991 to 2004, followed by a significant rise after 2005. The United States emerged as the leading country in publication volume. Harvard University was identified as a globally influential institution with 126 publications. Although a large author collaboration cluster and several smaller ones were formed, most collaborations between authors were relatively weak, with no high-density integrated academic network yet established. Pietsch Hubertus was the most prolific author, while Bae KT was the most highly co-cited author. The most highly cited journal was Radiology, with 2,384 citations. Co-occurrence analysis revealed that the top three keywords by frequency were “agent,” “CT,” and “image quality.” Keyword clustering analysis showed that the top three clusters were “gadolinium,” “gold nanoparticles,” and “image quality.” The timeline analysis indicated that clusters such as “gadolinium,” “gold nanoparticles,” “image quality,” and “material decomposition” exhibited strong temporal continuity, while the keyword with the highest burst value was “digital subtraction angiography” (19.38). Burst term trend analysis suggested that recent research has been focusing on areas like “deep learning,” “risk,” “radiation dosage,” and “iodine quantification.”

**Conclusion:**

This study is the first to systematically reveal the global trends, hotspots, frontiers, and development dynamics of iodine contrast agents in medical imaging through the use of CiteSpace and VOSviewer. It provides a novel perspective for understanding the role of iodine contrast agents in imaging and offers valuable insights for advancing global research in medical imaging.

## 1 Introduction

Iodinated contrast agents, also known as iodinated imaging agents, are iodine-containing contrast agents formed by iodine atoms combined with various compounds. Iodinated contrast agents are among the most commonly used drugs for angiography. As early as 2002, more than 75 million cases of intravascular iodinated contrast agent use were recorded globally (1).

Iodinated contrast agents enhance contrast in imaging modalities such as X-ray, CT, and MRI, making tissue structures and lesions more visible, thus assisting physicians in better diagnosing and assessing conditions. Clinically, iodinated contrast agents are commonly used in CT angiography, CT perfusion, and imaging of body cavities, joints, and the spinal cord (2–6). With their superior imaging capabilities, iodinated contrast agents can quickly and clearly delineate the contours of specific organs or tissues, proving highly effective and advantageous in the accurate diagnosis of tumors, detailed exploration of vascular diseases, and precise identification of inflammation (7). Moreover, they can be used to guide surgical procedures, catheter insertions, and other therapeutic operations, enhancing precision and helping doctors monitor changes in lesions during treatment (8, 9).

Bibliometric analysis, as an emerging and powerful tool, ingeniously integrates statistical methods with visualization techniques, opening new avenues for rapidly understanding the current state and future trends of specific disciplines or fields. This method excels in capturing critical nodes within a field while extracting academically valuable information from vast amounts of data. On the broad stage of bibliometrics, bibliometric software plays a pivotal role, with CiteSpace and VOSviewer standing out as favored tools among researchers (10). Although the application of iodinated contrast agents in medical imaging has increasingly become a research hotspot among global scholars, systematic visualization analysis of their application from a global perspective remains scarce. Therefore, this study aims to utilize CiteSpace and VOSviewer to conduct a detailed bibliometric analysis of relevant literature, exploring the application of iodinated contrast agents in medical imaging, and uncovering research hotspots, trends, and frontiers, with the goal of providing insights for future research directions.

## 2 Materials and methods

### 2.1 Inclusion and exclusion criteria

The Web of Science (WOS) Core Collection was used as the literature retrieval database, with citation indices including the Science Citation Index Expanded, Social Sciences Citation Index, and Emerging Sources Citation Index. The included literature had to meet the following criteria: (1) studies related to the application of iodinated contrast agents in medical imaging; (2) the literature provided basic information such as author, country, institution, keywords, and references for research analysis; (3) the publication period ranged from the inception of the database to July 31, 2024; and (4) no language restrictions were applied. The exclusion criteria were: (1) conference abstracts; (2) letters; (3) editorial materials; (4) corrections; (5) conference proceedings; and (6) duplicate publications.

### 2.2 Literature retrieval

**Figure 1:**
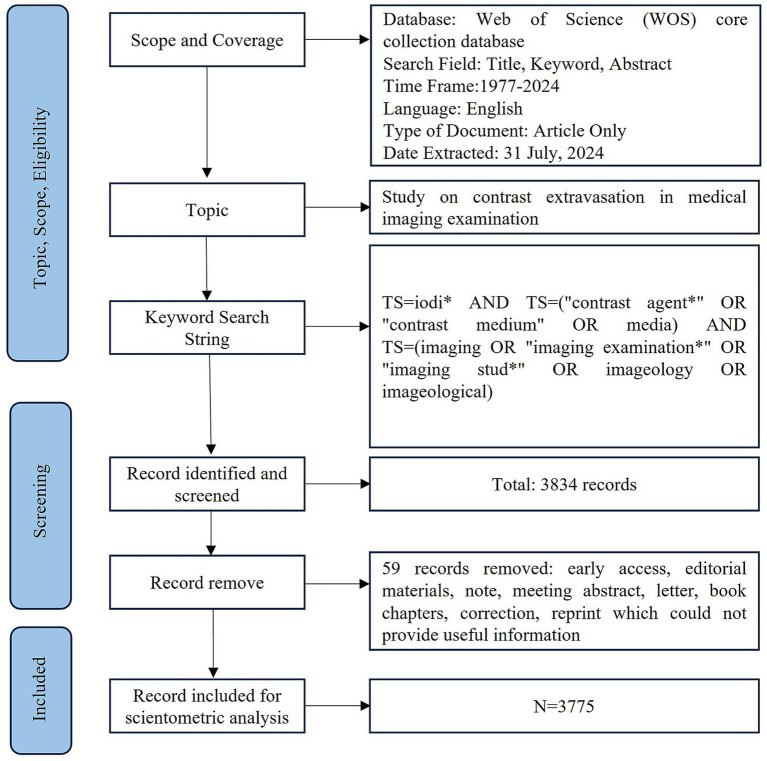
The research flow chart.

The search strategy was [TS = iodi* AND TS = (“contrast agent*” OR “contrast medium” OR media) AND TS = (imaging OR “imaging examination*” OR “imaging stud*” OR imageology OR imageological)]. The search period was from January 1, 1977, to July 31, 2024, yielding 3,834 relevant publications. After excluding 59 documents that did not provide the necessary information for bibliometric analysis (such as author country, author institution, keywords, and citations), including early access articles, conference papers, letters, editorial materials, and book chapters, a total of 3,775 documents were identified. All retrieved articles were imported into CiteSpace for duplicate checking and preprocessing, with no duplicates found. Ultimately, 3,775 publications were included in this study. The literature retrieval and screening process is shown in Figure 1.

### 2.3 Ethical approval

This study did not require ethical approval as all data were obtained from publicly available published literature.

### 2.4 Statistical analysis

CiteSpace 6.3, VOSviewer 1.6, and Excel 2016 were utilized to identify journals, institutions, countries, keyword co-occurrences, co-cited references, and research trends, providing a visual representation of the research landscape. Keyword and subject term co-occurrence analysis was used to uncover research hotspots in the field. Co-citation analysis and burst term detection were employed to explore the dynamic frontiers of research. Cluster analysis of keywords and subject terms, combined with a timeline view, was used to display the progression of research trajectories. Additionally, collaboration network diagrams of authors and institutions were used to identify key figures and core teams in the field, while the co-occurrence network of journals revealed the intersections and integrations between different disciplines.

## 3 Results

### 3.1 Analysis of annual publication volume

**Figure 2:**
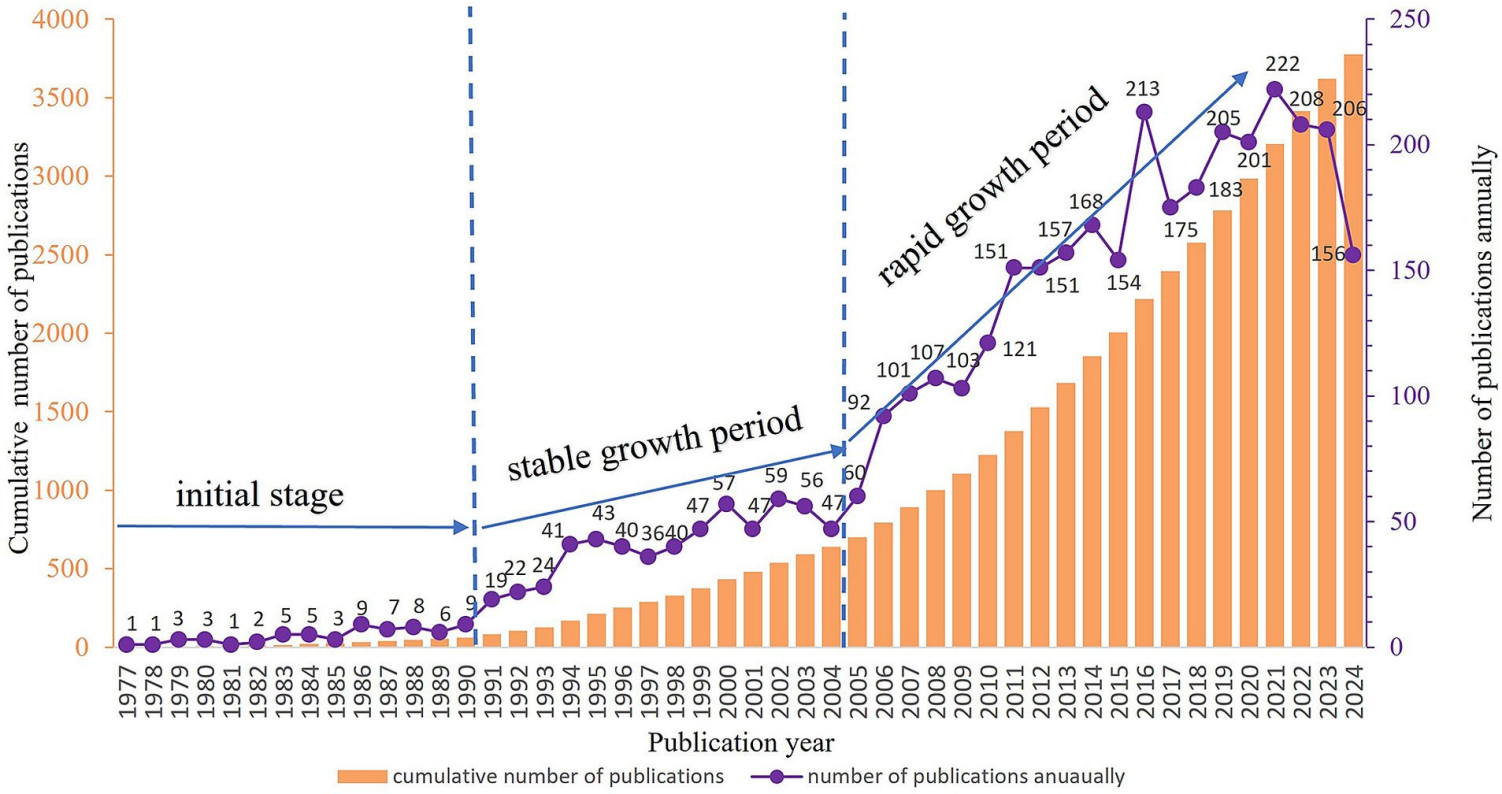
Annual and cumulative trend of publications.

A total of 3,775 valid publications were included in this study. The first research paper in this field indexed in the Web of Science Core Collection was published in 1977 (11). From 1977 to 1990, research on iodinated contrast agents in medical imaging examinations was in the preliminary exploration stage, with relatively few publications. Between 1991 and 2004, the field experienced a period of slow development, with a slight annual increase in the number of publications. After 2005, the number of publications entered a rapid growth phase, with a significant surge in publication volume (Figure 2).

### 3.2 Collaboration network analysis

#### 3.2.1 Analysis of national publication volume and co-occurrence network

**Figure 3:**
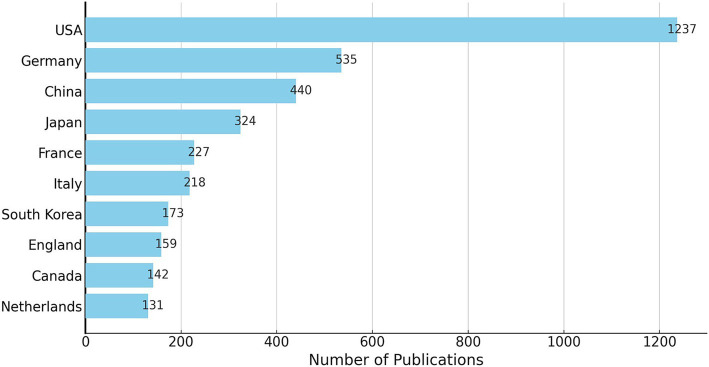
The distribution of the top 10 countries that had the most publications.

**Figure 4:**
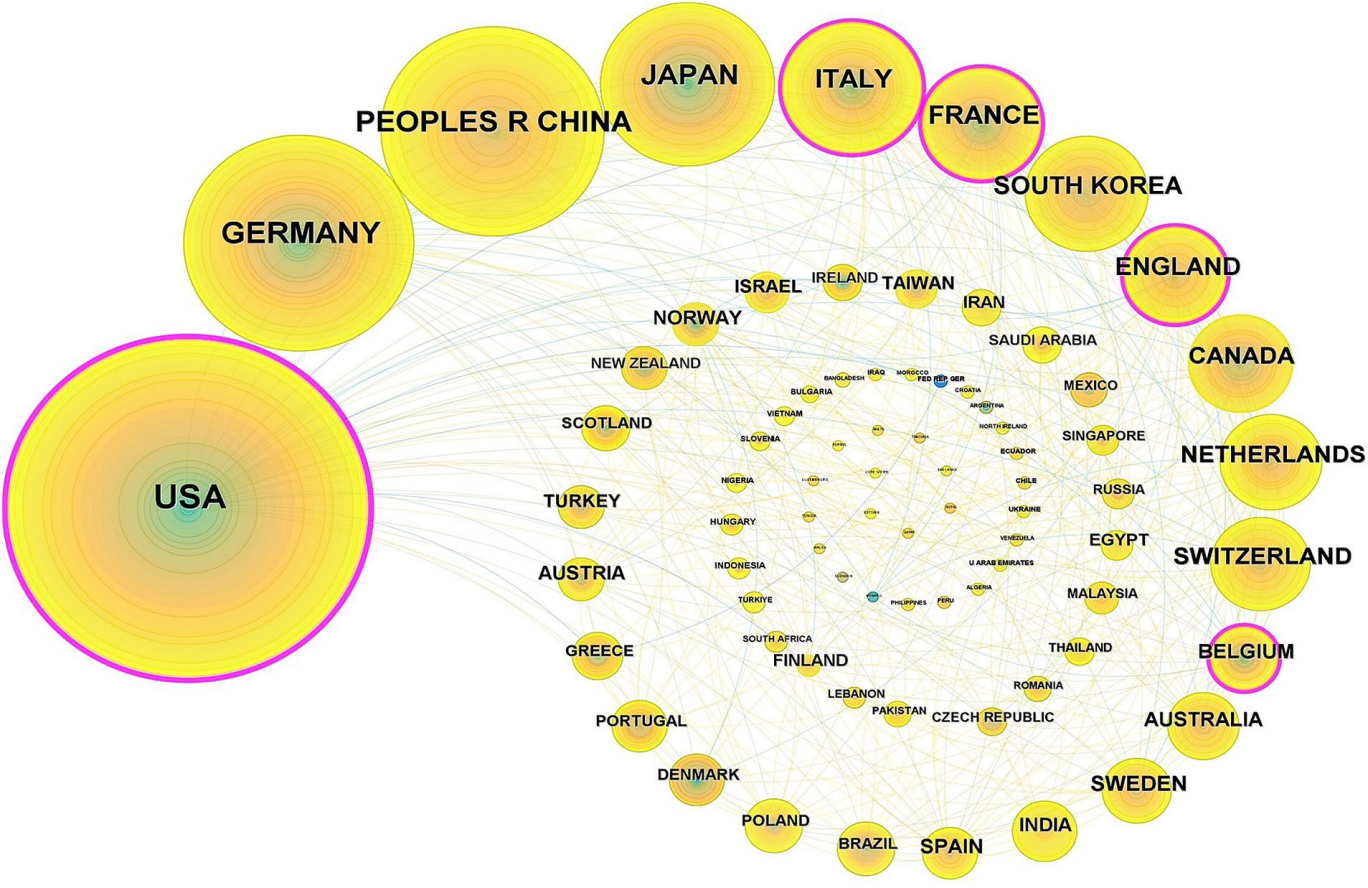
Countries co-occurrence analysis (Each node represents a distinct country. The size of each node corresponds to the number of papers published by that country. The lines connecting the nodes illustrate the collaborative relationships between countries, with the thickness of the lines indicating the strength of these collaborations).

A total of 81 countries have conducted research on the application of iodinated contrast agents in medical imaging. The United States had the highest number of publications, accounting for one-third of the total (1,237 papers), followed by Germany (535 papers), China (440 papers), and Japan (324 papers). Together, these four countries accounted for two-thirds of the total publications. Figure 3 shows the top 10 countries by publication volume. In this research field, countries with high betweenness centrality were primarily the United States (0.51), England (0.19), Italy (0.17), Belgium (0.14), France (0.11), Germany (0.10), and China (0.10), while the centrality of other countries was below 0.10, as shown in Figure 4.

#### 3.2.2 Analysis of institutional publication volume and collaboration network

**Figure 5:**
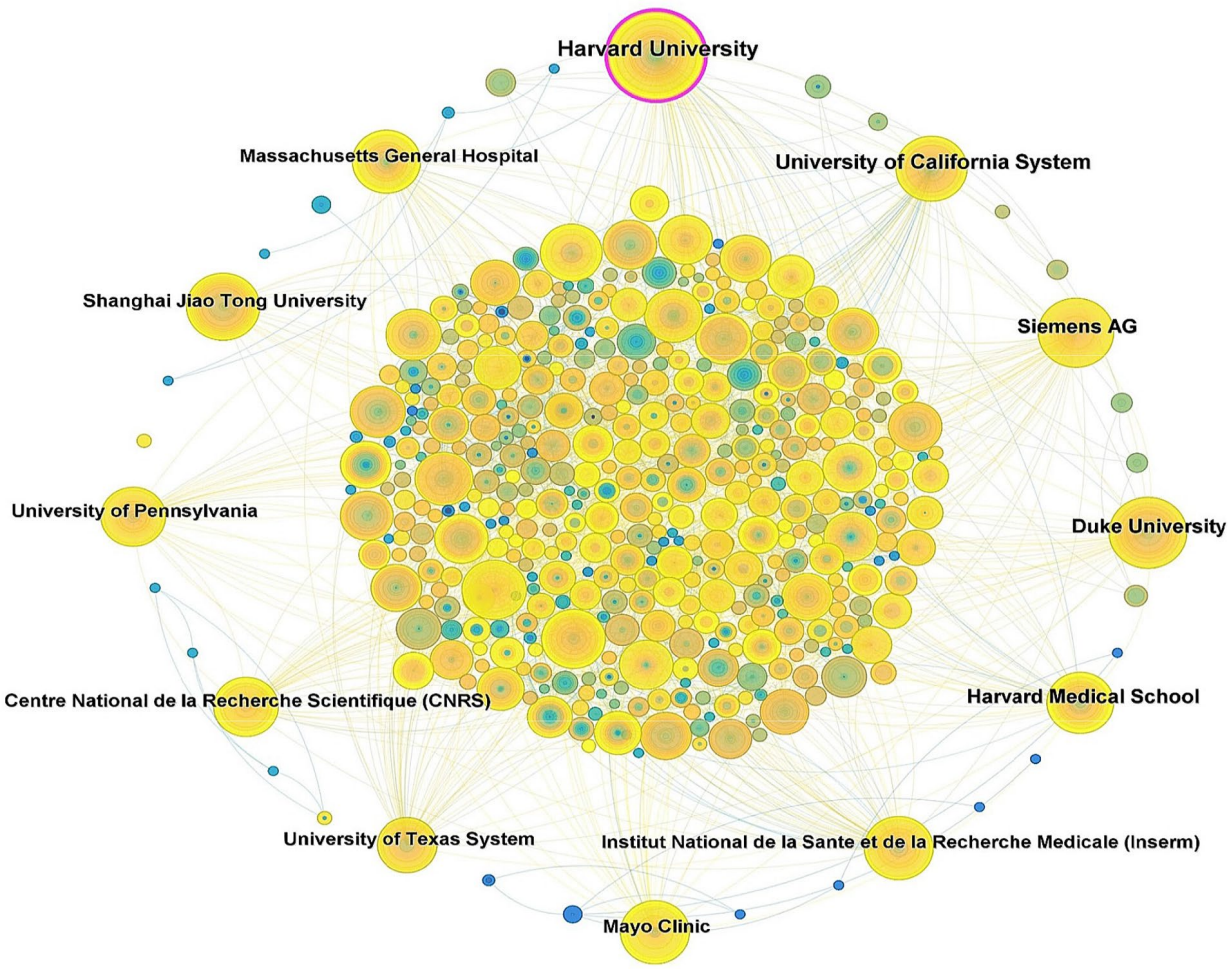
Cooperative network diagram of research institutions (Each node represents a distinct institution. The size of each node indicates the number of papers published by that institution. The lines connecting the nodes represent the collaborative relationships between institutions, with the thickness of the lines denoting the strength of these collaborations).

Globally, 671 research institutions have conducted studies on the application of iodinated contrast agents in medical imaging. Figure 5 illustrates the collaboration network among these institutions, and the top 10 institutions by publication volume are listed in Table 1. The three institutions with the highest publication volumes are Harvard University (126 papers), the University of California System (104 papers), and Siemens AG (78 papers). In terms of institutional collaboration centrality, Harvard University is the only institution with a betweenness centrality exceeding 0.10.

**Table 1:** Top 10 institutions in terms of number of publications and intermediary centrality.

Rank	Publication		Centrality	
	Number of publication	Institution	Centrality	Institution
1	126	Harvard University	0.10	Harvard University
2	104	University of California System	0.09	Siemens AG
3	78	Siemens AG	0.08	University of Texas System
4	76	Duke University	0.07	University of California System
5	72	Harvard Medical School	0.06	Institut National de la Sante et de la Recherche Medicale (Inserm)
6	67	Massachusetts General Hospital	0.05	General Electric
7	65	Institut National de la Sante et de la Recherche Medicale (Inserm)	0.05	Assistance Publique Hopitaux Paris (APHP)
8	65	Mayo Clinic	0.04	Technical University of Munich
9	58	University of Texas System	0.04	Philips
10	57	Center National de la Recherche Scientifique (CNRS)	0.04	University System of Ohio

#### 3.2.3 Author collaboration network analysis and co-cited author analysis

**Figure 6:**
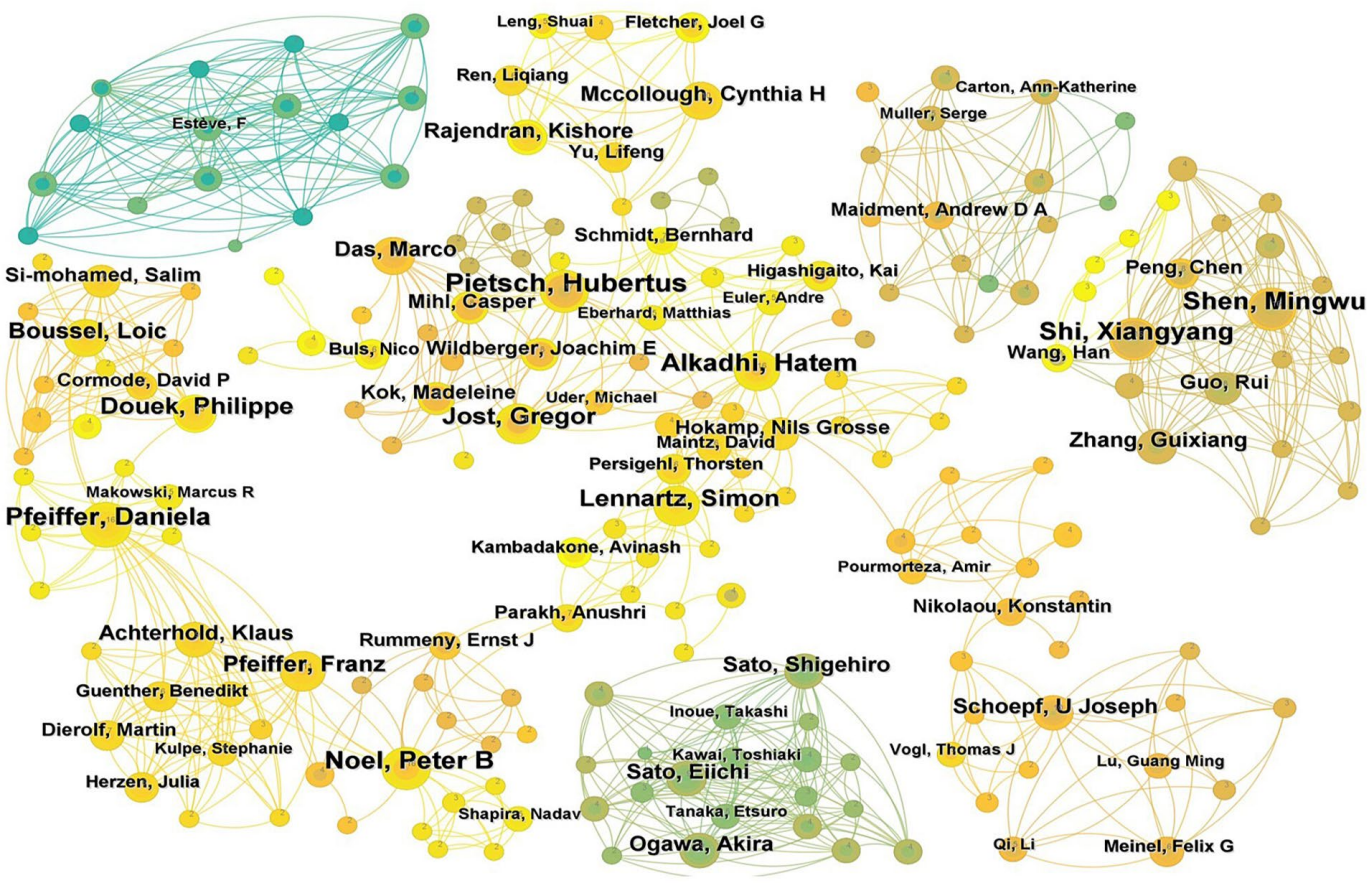
Author cooperation network map (Each node represents a distinct author. The size of each node corresponds to the number of papers published by that author. The lines connecting the nodes indicate the collaborative relationships between authors, with the thickness of the lines denoting the strength of these collaborations. Different colors represent various author collaboration clusters).

To identify influential authors in the field of iodinated contrast agents in medical imaging, we mapped the collaborative network of 1,395 authors (Figure 6), where each node represents an author and different colors signify various collaboration clusters. Due to the relatively weak collaboration among many authors, the figure only displays the collaboration relationships of the 206 authors with the closest partnerships.

Table 2 lists the top 10 authors by publication volume and the top 10 co-cited authors. The results show that the most prolific author is Pietsch Hubertus (17 papers), followed by Shi Xiangyang (16 papers), Noel Peter B (16 papers), Alkadhi Hatem (16 papers), Shen Mingwu (16 papers), and Pfeiffer Daniela (16 papers). The top three co-cited authors are Bae KT (262 citations), McCollough CH (165 citations), and Davenport MS (140 citations), indicating that these scholars’ contributions have been widely recognized within the field.

**Table 2:** Top 10 authors and co-cited authors.

Rank	Author	Publications (*n*)	First Publication Year	Co-cited Author	Co-citations (*n*)	First Publication Year
1	Pietsch Hubertus	17	2012	Bae KT	262	2003
2	Shi Xiangyang	16	2010	McCollough CH	165	2004
3	Noel Peter B	16	2014	Davenport MS	140	2014
4	Alkadhi Hatem	16	2011	Thomsen HS	136	2006
5	Shen Mingwu	16	2010	Lusic H	125	2014
6	Pfeiffer Daniela	16	2019	Katayama H	123	1994
7	Jost Gregor	14	2013	Johnson TRC	123	2008
8	Douek Philippe	13	2017	Cormode DP	121	2011
9	Lennartz Simon	13	2019	Mccullough PA	120	2006
						
10	Badea CT	12	2012	Hainfeld JF	119	2010

### 3.3 Co-cited literature analysis

The 3,775 publications collectively cited 9,105 references. Table 3 shows the top 10 most frequently co-cited papers (12–21). The most highly co-cited paper is Lusic H’s 2013 paper titled “X-ray-Computed Tomography Contrast Agents,” which provides an in-depth discussion on the application of iodinated contrast agents in X-ray CT, particularly their ability to enhance soft tissue visibility in diagnostic and therapeutic monitoring. It also highlights the safety and efficacy improvements achieved through chemical and nanotechnology advancements in contrast agents (12). This paper has been cited a total of 51 times. The publication dates of these highly cited articles are primarily concentrated in the past decade.

**Table 3:** Top 10 most co-cited research articles.

ID	Title	Journal	Publication year	First author	Co-citations (*n*)	Centrality
1	X-ray-Computed Tomography Contrast Agents	Chemical Reviews	2013	Lusic H	51	0.01
2	Dual- and Multi-Energy CT: Principles, Technical Approaches, and Clinical Applications	Radiology	2015	McCollough CH	50	0.06
3	Photon-counting CT: Technical Principles and Clinical Prospects	Radiology	2018	Willemink MJ	37	0.01
4	Nano-Sized CT Contrast Agents	Advanced Materials	2013	Lee N	31	0.00
5	Post-contrast acute kidney injury—Part 1: Definition, clinical features, incidence, role of contrast medium and risk factors	European Radiology	2018	van der Molen AJ	31	0.00
6	Use of Intravenous Iodinated Contrast Media in Patients with Kidney Disease: Consensus Statements from the American College of Radiology and the National Kidney Foundation	Radiology	2020	Davenport MS	31	0.01
7	Accuracy of iodine quantification using dual energy CT in latest generation dual source and dual layer CT	European Radiology	2017	Pelgrim GJ	29	0.00
8	Photon-counting CT for simultaneous imaging of multiple contrast agents in the abdomen: An *in vivo* study	Medical Physics	2017	Symons R	28	0.01
9	A High-Performance Ytterbium-Based Nanoparticulate Contrast Agent for *In Vivo* X-Ray Computed Tomography Imaging	Angewandte Chemie-International Edition	2012	Liu YL	27	0.02
10	Nanoparticle contrast agents for computed tomography: a focus on micelles	Contrast Media & Molecular Imaging	2014	Cormode DP	27	0.02

### 3.4 Co-cited journal analysis

The 9,105 co-cited references were published across 1,210 journals. Table 4 presents the top 10 most frequently cited journals, indicating that these journals have published a significant number of studies on the application of iodinated contrast agents in medical imaging. The journal with the highest number of citations is Radiology, with a total of 2,384 citations.

**Table 4:** Top 10 journals according to co-citation counts.

Journal	Impact factor (2023)	Count	Centrality
Radiology	12.1	2,384	0.02
Am J Roentgenol	4.7	1807	0.02
Eur Radiol	4.7	1,526	0.02
Invest Radiol	7.0	1,461	0.02
Eur J Radiol	3.2	1,027	0.01
Brit J Radiol	1.8	852	0.04
Med Phys	3.2	846	0.02
J Comput Assist Tomo	1.0	767	0.02
Acad Radiol	3.8	731	0.02
Phys Med Biol	3.3	686	0.03

### 3.5 Keyword analysis

#### 3.5.1 Co-occurrence analysis

**Figure 7:**
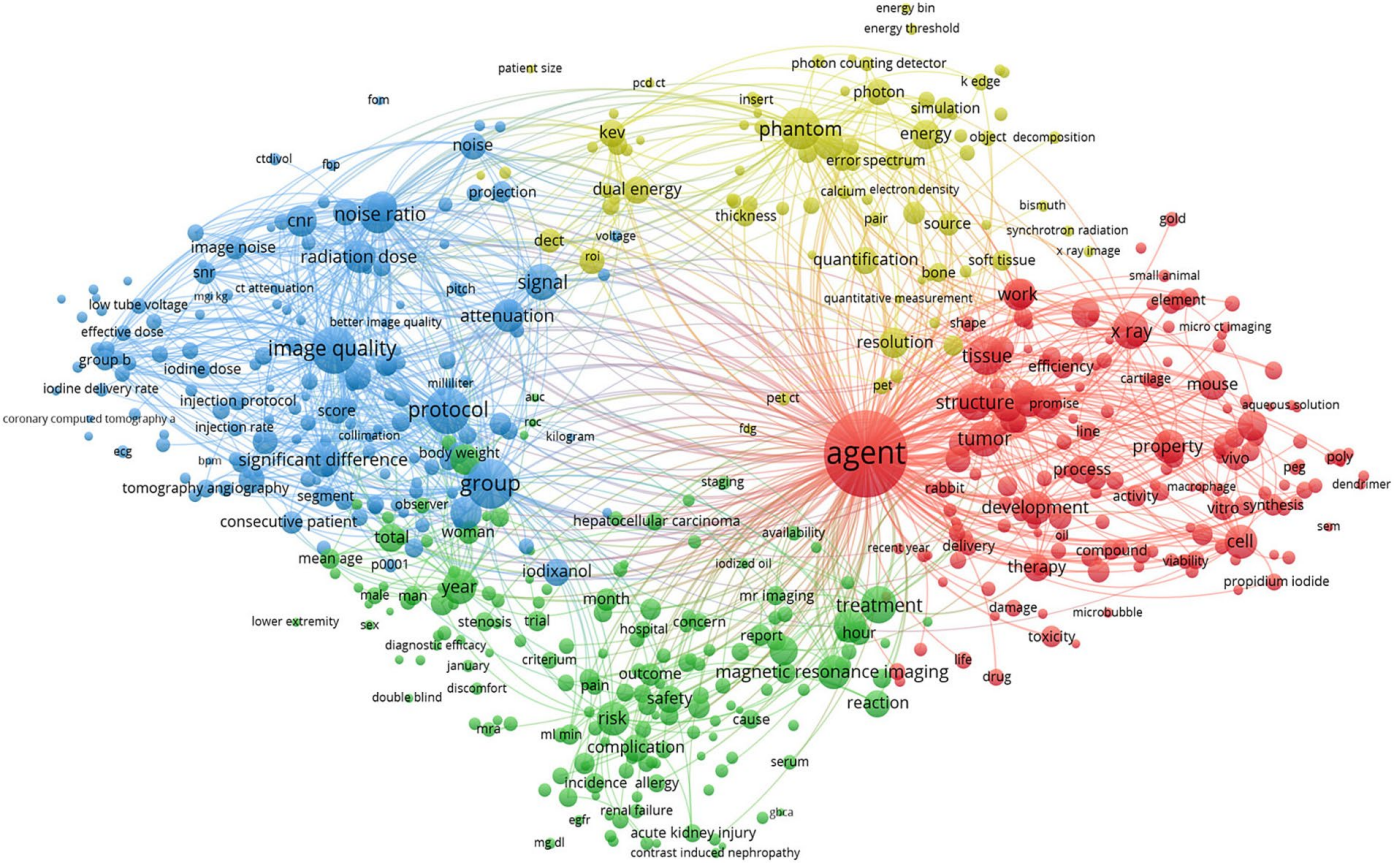
Keywords contribution analysis (Each node represents a distinct keyword. The size of each node reflects the frequency of that keyword. The lines connecting the nodes illustrate the relationships between keywords, while different colors represent various keyword clusters).

Keywords serve as summaries and abstractions of the main content and key information of publications. By analyzing the keywords, one can identify research hotspots and directions within a given field. This study identified a total of 76,850 keywords. Using VOSviewer, a co-occurrence analysis was performed on the 580 keywords that appeared 20 or more times (Figure 7). Table 5 shows the top 20 most frequently occurring keywords, with the top 5 being “agent,” “CT,” “image quality,” “angiography,” and “enhancement.”

**Table 5:** Top 20 high-frequency keywords.

Rank	Keywords	Count	Centrality	First Publication Year	Rank	Keywords	Count	Centrality	First Publication Year
1	Agent	1,348	0.14	1991	11	*in vivo*	111	0.03	2002
2	CT	1,147	0.13	1991	12	Liver	109	0.07	1992
3	Image quality	261	0.03	2004	13	Gold nanoparticles	105	0.02	2010
4	Angiography	244	0.05	1991	14	Risk	104	0.01	2007
5	Enhancement	159	0.05	1993	15	Acute kidney injury	98	0.01	2012
6	CT angiography	147	0.04	2001	16	Safety	97	0.02	1994
7	Diagnosis	143	0.08	1994	17	Iodine	96	0.03	1997
8	Induced nephropathy	126	0.02	2006	18	Computed tomography angiography	94	0.01	2007
9	Dual energy CT	114	0.01	2011	19	Digital subtraction angiography	93	0.04	1993
10	Cancer	113	0.05	1991	20	MRI	88	0.03	2002

#### 3.5.2 Cluster analysis

**Figure 8:**
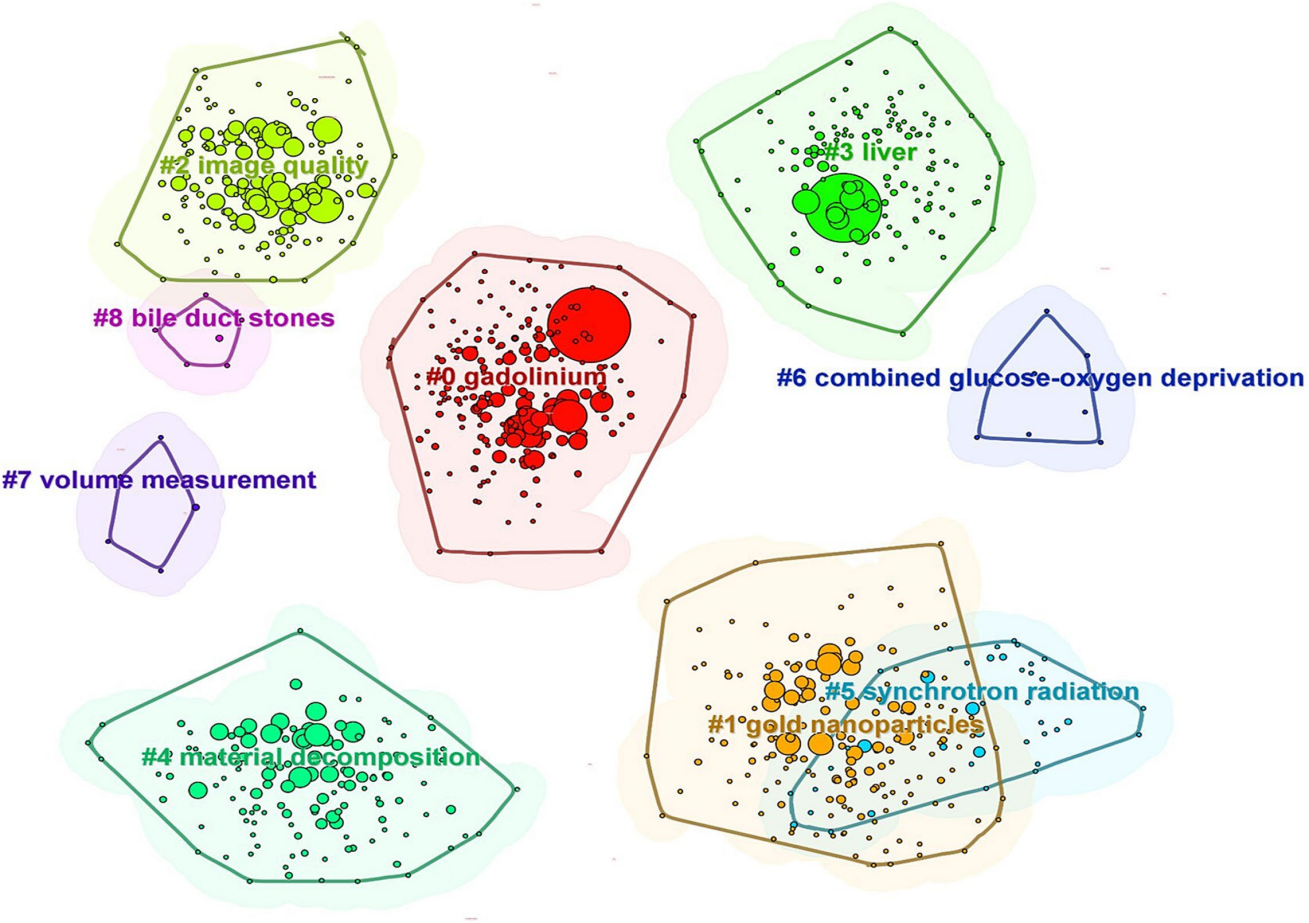
Network map of keywords (Each node represents a distinct keyword. Different colors signify various keyword clusters, with datasets within the same cluster exhibiting maximum similarity and datasets in different clusters demonstrating minimal similarity).

Through the cluster analysis of keywords (Figure 8), nine distinct clusters were identified. The top five largest clusters were labeled as “gadolinium,” “gold nanoparticles,” “image quality,” “liver,” and “material decomposition.” These clusters reflect the major thematic areas of research in the application of iodinated contrast agents in medical imaging.

#### 3.5.3 Timeline and burst term analysis

**Figure 9:**
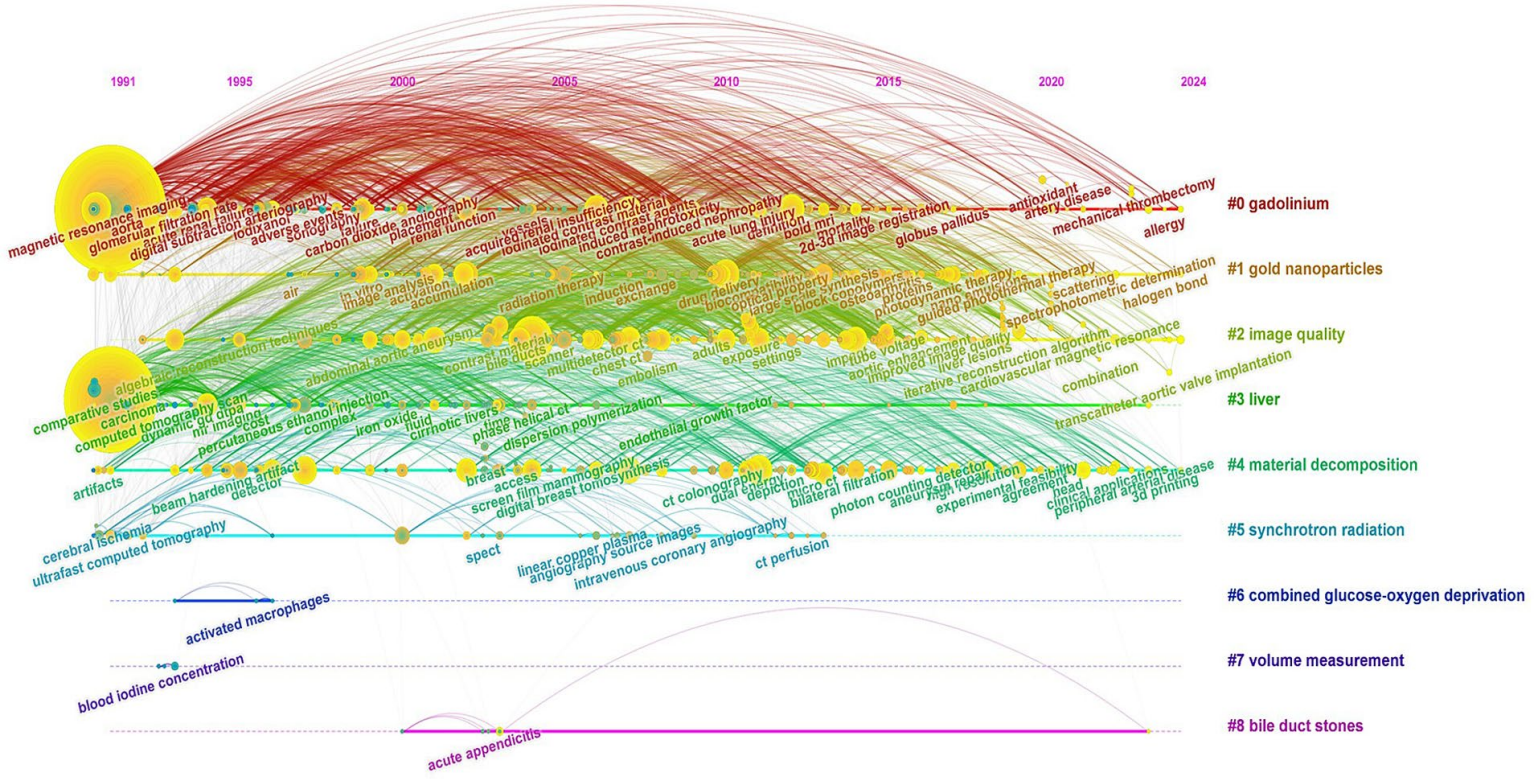
Keyword timeline distribution (Each node represents a distinct keyword, and the size of each node reflects the frequency of that keyword. Keywords within the same cluster are positioned on the same horizontal line, while different colors denote different keyword clusters).

A timeline visualization was created for the keyword clusters to analyze how keywords have evolved over time (Figure 9). The first high betweenness centrality keywords to appear were “agent” and “CT,” which belong to the “gadolinium” and “gold nanoparticles” clusters, respectively. The timeline shows that clusters such as “gadolinium,” “gold nanoparticles,” “image quality,” and “material decomposition” demonstrate strong temporal continuity and have maintained high attention over time. In contrast, clusters such as “combined glucose–oxygen deprivation,” “volume measurement,” and “bile duct stones” exhibit weaker temporal continuity, indicating lower levels of ongoing research interest.

**Figure 10:**
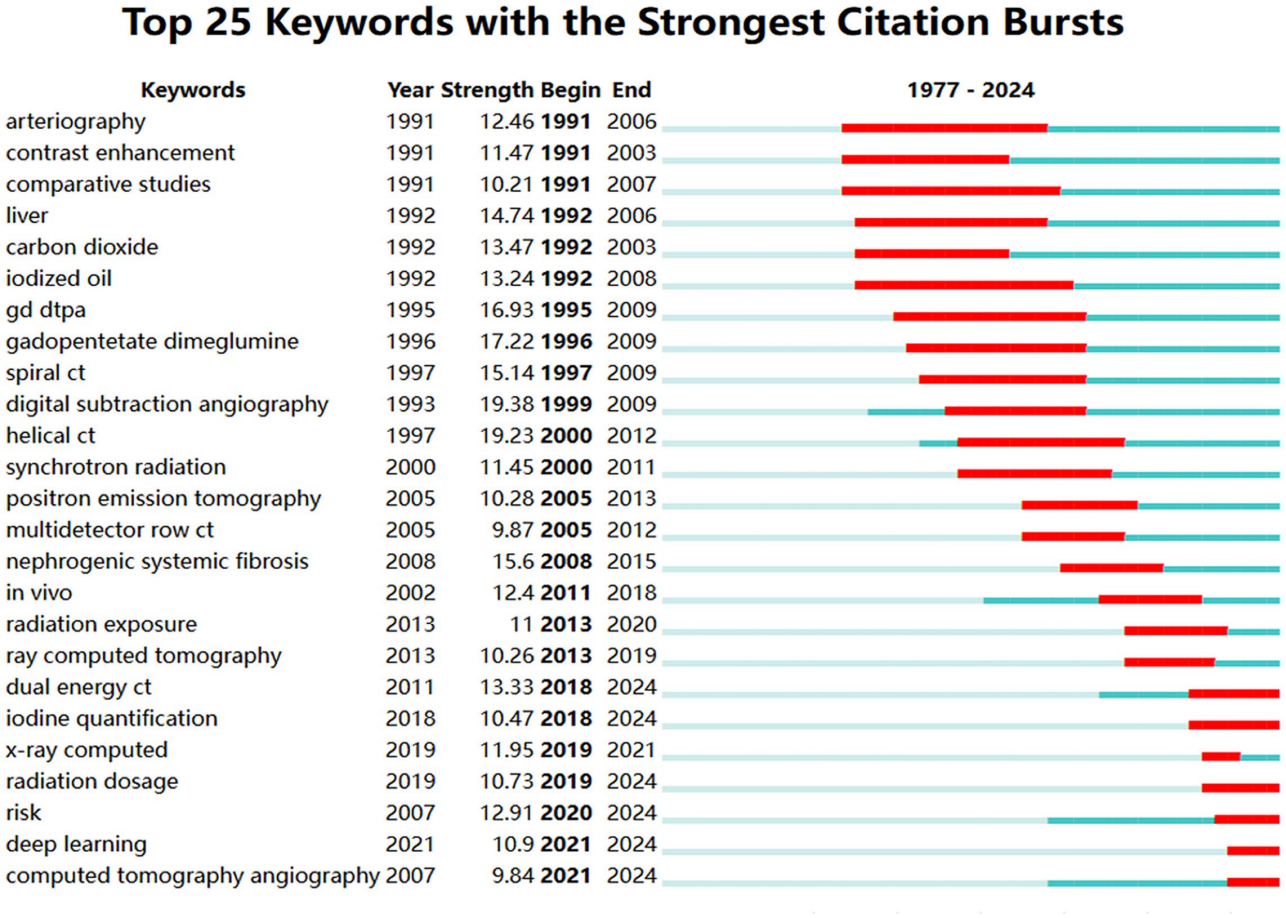
Map of top 25 keywords with the strongest citation bursts (The red line represents the time period in which the keyword appears. Strength represents the value of burst detection, where a higher value indicates a higher credibility of the burst word’s appearance during a specific period).

Figure 10 presents the top 25 most prominent burst terms related to the application of iodinated contrast agents in medical imaging. The burst term with the highest strength in this study was “digital subtraction angiography” (19.38). Analyzing the evolution of burst terms reveals that, starting in 1991, terms such as “arteriography,” “contrast enhancement,” “carbon dioxide,” and “iodized oil” exhibited strong burst strengths, indicating that these contrast agent-related technologies received widespread attention and application in the early 1990s. Beginning in 1995, keywords related to gadolinium-based contrast agents, such as “GD-DTPA” and “gadopentetate dimeglumine,” showed high burst strengths, continuing through the late 2000s. This reflects the growing comparison between gadolinium and iodinated contrast agents in clinical applications during that period. In 1997, terms like “spiral CT” (19.23) and “helical CT” (15.14) began to burst with significant strength, indicating the rapid development of novel CT imaging technologies during this time. In the late 2000s and 2010s, terms such as “synchrotron radiation” and “dual energy CT” started to burst, reflecting the broader adoption of imaging techniques like synchrotron radiation imaging and dual-energy CT in medical imaging. The keyword “nephrogenic systemic fibrosis” began to burst in 2008 and continued through 2015, indicating heightened concern over nephrogenic systemic fibrosis associated with gadolinium- and iodine-based contrast agents during that period. Since 2018, keywords such as “iodine quantification,” “radiation dosage,” and “risk” have emerged as burst terms, highlighting the growing focus on the exposure risks of iodinated contrast agents and radiation doses in the field of medical imaging. Most recently, since 2021, “deep learning” has emerged as a burst keyword, indicating increasing attention and recognition of the intersection between artificial intelligence and medical imaging.

## 4 Discussion

This study is the first to systematically present the global research status, hotspots, trends, and frontiers in the application of iodinated contrast agents in medical imaging over the past 40 years through bibliometric analysis using CiteSpace and VOSviewer. The detailed visualization maps provided in this study offer a comprehensive understanding of the application of iodinated contrast agents in medical imaging.

The first publicly available research in this field was published in 1977. From 1977 to 1990, research on iodinated contrast agents in medical imaging was in its exploratory phase, with a relatively small number of academic publications. Between 1991 and 2004, the field experienced slow development, with a slight annual increase in publication volume, likely driven by the introduction of new low-osmolar iodinated contrast agents, advancements in injection techniques, ongoing developments in imaging technologies, and the rise of multimodal fusion imaging. Since 2005, there has been a rapid growth phase, with a significant increase in publication volume. This trend has persisted despite the disruptions caused by the COVID-19 pandemic, which led to the suspension or reduction of many research projects (22–25). The primary reasons for this trend may include the global increase in chronic diseases and the growing need for radiological information in medical diagnostics. Techniques such as PET/CT play a significant role in clinical practice worldwide (26). Consequently, this has significantly expanded the use of iodinated contrast agents as a common drug in radiology over the past two decades (27).

Globally, 81 countries have conducted research on the application of iodinated contrast agents in medical imaging, with the United States being the leader in this field, accounting for one-third of the total publications. The highest betweenness centrality in this research field is concentrated in the United States, England, Italy, Belgium, and France, indicating close international collaborations among these countries. Although Germany and China rank second and third in publication volume, their betweenness centrality is not as high, suggesting that the research quality or utility in these countries may be lower, with fewer international collaborations and less widespread dissemination of their research findings. Institutional publication volume and collaboration network analyses reveal that Harvard University leads the field in publication volume and has the highest betweenness centrality, indicating its central role in this research domain. Siemens AG follows closely behind Harvard University in centrality, highlighting the significant role of medical equipment manufacturers. Additionally, other medical equipment giants such as General Electric and Philips are also among the top 10 in centrality, further underscoring the substantial contributions of these companies to the application of iodinated contrast agents in medical imaging. In terms of author and co-cited author analysis, leading authors like Pietsch Hubertus, Shi Xiangyang, and Noel Peter B are prominent in terms of research output. However, the research group led by Shi Xiangyang has relatively few international collaborations with other research groups. Co-citation analysis shows that the work of authors like Bae KT, McCollough CH, and Davenport MS has received widespread attention, reflecting key research areas such as the application of iodinated contrast agents in CT imaging (28, 29), radiation dose safety (30, 31), and renal function assessment (32, 33). In the past decade, the research of authors like Lusic H, Johnson TRC, and Cormode DP has gained prominence, indicating emerging research areas in molecular imaging (34, 35), dual-energy CT technology (36–38), and nanotechnology (39–41).

Through keyword co-occurrence and cluster analysis, the primary themes and focus areas of research can be better understood. This study identified the four major themes in the application of iodinated contrast agents in medical imaging: (1) the “gadolinium” field, represented by keywords such as “agent,” “angiography,” and “acute kidney injury”; (2) the “gold nanoparticles” field, represented by keywords like “*in vivo*,” “MRI,” and “cancer”; (3) the “image quality” field, represented by keywords such as “enhancement,” “CT angiography,” and “iterative reconstruction”; and (4) the “liver” field, represented by keywords like “CT,” “diagnosis,” and “spiral CT.” These clusters all demonstrate strong temporal continuity when viewed on a timeline.

To further explore the development trends and turning points in the application of iodinated contrast agents in medical imaging, and to identify research directions with potential value, this study used burst term analysis to highlight terms that appeared or were frequently used during specific periods. The highest burst term identified in this study was “digital subtraction angiography” (19.38). The significant attention to “digital subtraction angiography” as a widely used imaging technique for vascular examination and interventional therapy was evident from 1999 to 2009. The evolution of burst terms over time shows that in the early 1990s, foundational technologies related to contrast agents, such as “agent” and “arteriography,” were the focus of considerable attention. Starting in 1995, gadolinium-based contrast agents became a research hotspot, with comparative studies between gadolinium and iodinated contrast agents in clinical applications being a major focus (42). In the late 1990s and early 2000s, new CT imaging technologies like “spiral CT” developed rapidly. In the late 2000s and 2010s, new developments in imaging technologies such as “synchrotron radiation” and “dual energy CT” gained attention. From 2008 to 2015, there was significant concern over the issue of “nephrogenic systemic fibrosis” caused by contrast agents. In the late 2010s, radiation dose and exposure became key issues. Recently, the cross-application of artificial intelligence technologies like “deep learning” in medical imaging has garnered increasing attention (43, 44). These research hotspots reflect important progress and trends in the application of contrast agents, the development of new imaging technologies, safety assessments, and the application of artificial intelligence in this field. The aging global population and the further spread of chronic diseases will have a profound impact on the development of iodinated contrast agents in medical imaging. The future directions in this field are likely to focus on lower radiation doses, more precise imaging, and lower toxicity contrast agents. This will create opportunities for innovations and advancements in nanotechnology, artificial intelligence, and CT technology. Additionally, closer global academic collaboration and exchange will facilitate the dissemination and development of optimal technologies, advancing the application of iodinated contrast agents in medical imaging to new heights.

## 5 Limitations

This study has certain limitations that must be acknowledged. First, due to compatibility with citation data formats and ensuring high-quality citation analysis, this study is based solely on the Web of Science Core Collection database. Other important databases such as PubMed, Scopus, or IEEE Xplore were not included, which may result in incomplete coverage of this research field. Second, bibliometrics cannot fully and accurately reflect the actual quality and impact of academic papers, as citations can be influenced by various factors such as academic environment, norms, and evaluation mechanisms. Third, while this study analyzed collaboration networks among countries, institutions, and authors, it did not delve deeply into the actual influence and research quality behind these collaborations. Lastly, although CiteSpace excels in generating and visualizing scientific knowledge maps, it may not be as powerful as other specialized tools for deeper data analysis, such as text mining or sentiment analysis. Similarly, while VOSviewer offers basic visualization options, it may have some limitations in customization and personalization, making it challenging for users to fully tailor the visualizations to their specific needs, potentially rendering the results less intuitive or aligned with research objectives.

## 6 Conclusion

Using bibliometric methods, this study provides the first comprehensive and systematic overview of the progress, current status, trends, hotspots, and frontiers in the application of iodinated contrast agents in medical imaging. Since the first report on the application of iodinated contrast agents in medical imaging appeared in the Web of Science Core Collection data in 1977, the field has continuously evolved over the past 40 years. Globally, several small author collaboration clusters and one large collaboration cluster have formed, but there remains significant room for collaboration among scholars, institutions, and countries. Emerging topics such as “deep learning,” “risk,” “radiation dosage,” and “iodine quantification” are likely to become future research frontiers and directions, requiring scholars’ close attention. This study not only provides a new perspective on the application and development of iodinated contrast agents in medical imaging but is also expected to offer new insights and references for future medical imaging research.

## Data Availability

The original contributions presented in the study are included in the article/supplementary material, further inquiries can be directed to the corresponding author.

## References

[1] Christiansen C. X-ray contrast media—an overview. Toxicology. (2005) 209:185–7. doi: 10.1016/j.tox.2004.12.020, PMID: 15767033

[2] Hammel J Birnbacher L Makowski MR Pfeiffer F Pfeiffer D. Absolute iodine concentration for dynamic perfusion imaging of the myocardium: improved detection of poststenotic is chaemic in a 3d-printed dynamic heart phantom. Eur Radiol Exp. (2022) 6:51. doi: 10.1186/s41747-022-00304-x, PMID: 36310190 PMC9618471

[3] Honkanen JT Turunen MJ Tiitu V Jurvelin JS Toyras J. Transport of iodine is different in cartilage and meniscus. Ann Biomed Eng. (2016) 44:2114–22. doi: 10.1007/s10439-015-1513-2, PMID: 26661617

[4] Inoue K Bashir MT Warner AL Ebrahimi R Neverova NV Currier JW. Cardiac electrical and structural changes after iodinated contrast media administration: a longitudinal cohort analysis. Thyroid. (2024) 34:1163–70. doi: 10.1089/thy.2024.0131, PMID: 39163054

[5] Karout L Kalra MK. Survey of ct radiation doses and iodinated contrast medium administration: an international multicentric study. Eur Radiol. (2024). doi: 10.1007/s00330-024-11017-7, PMID: 39181948

[6] Rajiah PS Dunning C Rajendran K Tandon YK Ahmed Z Larson NB. High-pitch multienergy coronary ct angiography in dual-source photon-counting detector ct scanner at low iodinated contrast dose. Investig Radiol. (2023) 58:681–90. doi: 10.1097/RLI.0000000000000961, PMID: 36822655 PMC10591289

[7] Sato Y Ishiyama M Nakano S Nakao M Mun M Ninomiya H. Ringlike peripheral increased iodine concentration for the differentiation of primary lung cancer and pulmonary metastases on contrast-enhanced dual-energy ct. AJR Am J Roentgenol. (2023) 220:828–37. doi: 10.2214/AJR.22.28654, PMID: 36629308

[8] Magee T. Imaging of the post-operative shoulder: does injection of iodinated contrast in addition to mr contrast during arthrography improve diagnostic accuracy and patient throughput? Skeletal Radiol. (2018) 47:1253–61. doi: 10.1007/s00256-018-2927-3, PMID: 29549380

[9] Berlyand Y Fraga JA Succi MD Yun BJ Lee AH Baugh JJ. Impact of iodinated contrast allergies on emergency department operations. Am J Emerg Med. (2022) 61:127–30. doi: 10.1016/j.ajem.2022.08.052, PMID: 36096014

[10] Chen C. An information-theoretic view of visual analytics. IEEE Comput Graph Appl. (2008) 28:18–23. doi: 10.1109/mcg.2008.2, PMID: 18240783

[11] Naidich TP Pudlowski RM Leeds NE Naidich JB Chisolm AJ Rifkin MD. The normal contrast-enhanced computed axial tomogram of the brain. J Comput Assist Tomogr. (1977) 1:16–29. doi: 10.1097/00004728-197701000-00004 308069

[12] Lusic H Grinstaff MW. X-ray-computed tomography contrast agents. Chem Rev. (2013) 113:1641–66. doi: 10.1021/cr200358s, PMID: 23210836 PMC3878741

[13] Cormode DP Naha PC Fayad ZA. Nanoparticle contrast agents for computed tomography: a focus on micelles. Contrast Media Mol Imag. (2014) 9:37–52. doi: 10.1002/cmmi.1551, PMID: 24470293 PMC3905628

[14] McCollough CH Leng S Yu L Fletcher JG. Dual- and multi-energy ct: principles, technical approaches, and clinical applications. Radiology. (2015) 276:637–53. doi: 10.1148/radiol.2015142631, PMID: 26302388 PMC4557396

[15] Liu Y Ai K Liu J Yuan Q He Y Lu L. A high-performance ytterbium-based nanoparticulate contrast agent for in vivo x-ray computed tomography imaging. Angew Chem Int Ed Eng. (2012) 51:1437–42. doi: 10.1002/anie.201106686, PMID: 22223303

[16] Symons R Krauss B Sahbaee P Cork TE Lakshmanan MN Bluemke DA. Photon-counting ct for simultaneous imaging of multiple contrast agents in the abdomen: an in vivo study. Med Phys. (2017) 44:5120–7. doi: 10.1002/mp.12301, PMID: 28444761 PMC5699215

[17] Pelgrim GJ van Hamersvelt RW Willemink MJ Schmidt BT Flohr T Schilham A. Accuracy of iodine quantification using dual energy ct in latest generation dual source and dual layer ct. Eur Radiol. (2017) 27:3904–12. doi: 10.1007/s00330-017-4752-9, PMID: 28168368 PMC5544802

[18] Davenport MS Perazella MA Yee J Dillman JR Fine D McDonald RJ. Use of intravenous iodinated contrast media in patients with kidney disease: consensus statements from the american college of radiology and the national kidney foundation. Radiology. (2020) 294:660–8. doi: 10.1148/radiol.2019192094, PMID: 31961246

[19] van der Molen AJ Reimer P Dekkers IA Bongartz G Bellin MF Bertolotto M. Post-contrast acute kidney injury—part 1: definition, clinical features, incidence, role of contrast medium and risk factors: recommendations for updated esur contrast medium safety committee guidelines. Eur Radiol. (2018) 28:2845–55. doi: 10.1007/s00330-017-5246-5 29426991 PMC5986826

[20] Lee N Choi SH Hyeon T. Nano-sized ct contrast agents. Adv Mater. (2013) 25:2641–60. doi: 10.1002/adma.201300081, PMID: 23553799

[21] Willemink MJ Persson M Pourmorteza A Pelc NJ Fleischmann D. Photon-counting ct: technical principles and clinical prospects. Radiology. (2018) 289:293–312. doi: 10.1148/radiol.2018172656, PMID: 30179101

[22] Sohrabi C Mathew G Franchi T Kerwan A Griffin M Soleil CDMJ. Impact of the coronavirus (covid-19) pandemic on scientific research and implications for clinical academic training—a review. Int J Surg. (2021) 86:57–63. doi: 10.1016/j.ijsu.2020.12.008, PMID: 33444873 PMC7833269

[23] Kisby G Seow JH van Schie G Phatouros CC Lam KV Muir T. The great contrast shortage of 2022-lessons learnt in Australia. J Med Imag Radiat Oncol. (2023) 67:475–81. doi: 10.1111/1754-9485.13538, PMID: 37199049

[24] Koeppel DR Boehm IB. Shortage of iodinated contrast media: status and possible chances—a systematic review. Eur J Radiol. (2023) 164:110853. doi: 10.1016/j.ejrad.2023.110853 37156181 PMC10155429

[25] Shakir MA Garratt KN Wimmer NJ. Impact of acist cvi contrast delivery system on iodinated contrast media administration and waste. J Invasive Cardiol. (2024):36. doi: 10.25270/jic/24.00150 38787925

[26] Evangelista L Cuocolo A Pace L Mansi L Del VS Miletto P. Performance of fdg-pet/ct in solitary pulmonary nodule based on pre-test likelihood of malignancy: results from the italian retrospective multicenter trial. Eur J Nucl Med Mol Imaging. (2018) 45:1898–907. doi: 10.1007/s00259-018-4016-1, PMID: 29736699

[27] England A Rawashdeh M Moore N Young R Curran G McEntee MF. More sustainable use of iodinated contrast media—why? Radiography. (2024) 30:74–80. doi: 10.1016/j.radi.2024.06.023 38991461

[28] Zhu J Wang Z Kim Y Bae SK Tao C Gong J. Analysis of contrast time-enhancement curves to optimise CT pulmonary angiography. Clin Radiol. (2017) 72:340.e9–340.e16. doi: 10.1016/j.crad.2016.11.018, PMID: 28027777

[29] Goshima S Kanematsu M Noda Y Kawai N Kawada H Ono H. Minimally required iodine dose for the detection of hypervascular hepatocellular carcinoma on 80-kvp ct. AJR Am J Roentgenol. (2016) 206:518–25. doi: 10.2214/AJR.15.15138, PMID: 26901007

[30] Wang J Qu M Duan X Takahashi N Kawashima A Leng S. Characterisation of urinary stones in the presence of iodinated contrast medium using dual-energy ct: a phantom study. Eur Radiol. (2012) 22:2589–96. doi: 10.1007/s00330-012-2532-0, PMID: 22865225 PMC3970240

[31] Ren L Zhou Z Ahmed Z Rajendran K Fletcher JG McCollough CH. Performance evaluation of single- and dual-contrast spectral imaging on a photon-counting-detector ct. Med Phys. (2024) 51:8034–46. doi: 10.1002/mp.17367, PMID: 39235343

[32] Davenport MS. More evidence of acute kidney injury from intravenous iodinated contrast material. Radiology. (2023) 307:e231181. doi: 10.1148/radiol.231181, PMID: 37278636

[33] Umakoshi H Nihashi T Takada A Hirasawa N Ishihara S Takehara Y. Iodinated contrast media substitution to prevent recurrent hypersensitivity reactions: a systematic review and meta-analysis. Radiology. (2022) 305:341–9. doi: 10.1148/radiol.220370, PMID: 35852428

[34] Freedman JD Ellis DJ Lusic H Varma G Grant AK Lakin BA. Dgemric and cect comparison of cationic and anionic contrast agents in cadaveric human metacarpal cartilage. J Orthop Res. (2020) 38:719–25. doi: 10.1002/jor.24511, PMID: 31687789 PMC7071952

[35] Stewart RC Patwa AN Lusic H Freedman JD Wathier M Snyder BD. Synthesis and pre-clinical characterization of a cationic iodinated imaging contrast agent (ca4+) and its use for quantitative computed tomography of ex vivo human hip cartilage. J Med Chem. (2017) 60:5543–55. doi: 10.1021/acs.jmedchem.7b00234, PMID: 28616978 PMC6408935

[36] Yel I Booz C D’Angelo T Koch V Gruenewald LD Eichler K. Standardization of dual-energy ct iodine uptake of the abdomen and pelvis: defining reference values in a big data cohort. Diagnostics. (2024) 14. doi: 10.3390/diagnostics14182051 PMC11431114 39335730

[37] Ota T Onishi H Fukui H Tsuboyama T Nakamoto A Honda T. Prediction models for differentiating benign from malignant liver lesions based on multiparametric dual-energy non-contrast ct. Eur Radiol. (2024). doi: 10.1007/s00330-024-11024-8, PMID: 39186105 PMC11836082

[38] Zhang S Simard M Lapointe A Filion É Campeau MP Vu T. Evaluation of radiation dose effect on lung function using iodine maps derived from dual-energy computed tomography. Int J Radiat Oncol Biol Phys. (2024) 120:894–903. doi: 10.1016/j.ijrobp.2024.04.069, PMID: 38705488

[39] Goodwill PW Saritas EU Croft LR Kim TN Krishnan KM Schaffer DV. X-space mpi: magnetic nanoparticles for safe medical imaging. Adv Mater. (2012) 24:3870–7. doi: 10.1002/adma.201200221, PMID: 22988557

[40] Pan D Schirra CO Wickline SA Lanza GM. Multicolor computed tomographic molecular imaging with noncrystalline high-metal-density nanobeacons. Contrast Media Mol Imag. (2014) 9:13–25. doi: 10.1002/cmmi.1571, PMID: 24470291 PMC4076970

[41] Inose T Kitamura N Takano-Kasuya M Tokunaga M Une N Kato C. Development of x-ray contrast agents using single nanometer-sized gold nanoparticles and lactoferrin complex and their application in vascular imaging. Colloids Surf B: Biointerfaces. (2021) 203:111732. doi: 10.1016/j.colsurfb.2021.111732, PMID: 33839472

[42] Spinosa DJ Kaufmann JA Hartwell GD. Gadolinium chelates in angiography and interventional radiology: a useful alternative to iodinated contrast media for angiography. Radiology. (2002) 223:326–7. doi: 10.1148/radiol.2232010742, PMID: 11997531

[43] Azarfar G Ko SB Adams SJ Babyn PS. Applications of deep learning to reduce the need for iodinated contrast media for ct imaging: a systematic review. Int J Comput Assist Radiol Surg. (2023) 18:1903–14. doi: 10.1007/s11548-023-02862-w 36947337

[44] Lee T Yoon JH Park JY Lee J Choi JW Ahn C. Deep learning-based iodine contrast-augmenting algorithm for low-contrast-dose liver ct to assess hypovascular hepatic metastasis. Abdom Radiol. (2023) 48:3430–40. doi: 10.1007/s00261-023-04039-0, PMID: 37704805

